# A novel prognostic model for predicting patient survival and immunotherapy responsiveness in hepatocellular carcinoma: insights into the involvement of T-cell proliferation

**DOI:** 10.1007/s12094-023-03363-7

**Published:** 2023-12-21

**Authors:** Shengjie Tang, Rui Sun, Kechao Tang, Xiang Wei, Ming Liu, Huabing Zhang

**Affiliations:** 1 The First Clinical Medical College, Xuzhou Medical University, Xuzhou, 221004 China; 2 Department of Biochemistry and Molecular Biology, Metabolic Disease Research Center, School of Basic Medicine, Anhui Medical University, Hefei, 230032 China; 3 Department of General Surgery, The First Affiliated Hospital of Anhui Medical University, Hefei, 230022 China

**Keywords:** Hepatocellular carcinoma, T cells, Immunotherapy, Prognosis

## Abstract

**Background:**

The cancer-associated biological mechanisms and the implementation of immunotherapy are heavily impacted by the activities of T cells, consequently influencing the effectiveness of therapeutic interventions. Nevertheless, the mechanistic actions of T-cell proliferation in response to immunotherapy and the overall prognosis of individuals diagnosed with hepatocellular carcinoma (HCC) remains insufficiently understood. The present work seeks to present a comprehensive analysis immune landscape in the context of HCC.

**Methods:**

To achieve this objective, both clinical data and RNA sequencing data were acquired from authoritative databases such as The Cancer Genome Atlas (TCGA) and Gene Expression Omnibus (GEO).

**Results:**

Through the utilization of consensus clustering techniques, distinct molecular subtypes associated with T-cell proliferation were delineated. Following this, seven genes of prognostic significance were identified via a combination of Cox and Lasso regression analyses. By integrating these genes into a prognostic signature, the predictive capability of the model was verified through an examination of internal and external datasets. Moreover, immunohistochemistry and qRT-PCR tests have verified the reliability of prognostic markers. Notably, the high-risk group exhibited elevated expression of immune checkpoint genes as well as higher benefit in terms of drug sensitivity testing, as determined by the Chi-square test (*P* < 0.001). The risk score derived from the prognostic signature depicted considerable efficacy in predicting the survival outcomes of HCC cases.

**Conclusions:**

Overall, prognostic markers may become valuable predictive tool for individuals diagnosed with HCC, allowing for the prediction of their prognosis as well as the assessment of their immunological condition and response to immunotherapy.

**Supplementary Information:**

The online version contains supplementary material available at 10.1007/s12094-023-03363-7.

## Introduction

Cancer remains a prominent global cause of mortality, as highlighted by the research conducted by Bray et al. [1]. The year 2020 saw the diagnosis of over 19 million new cancer cases worldwide, with around 10 million deaths attributed to cancers. Specifically, liver cancer accounted for over 900,000 new cases of liver cancer and about 800,000 deaths [2]. HCC is the prevailing form of liver cancer, constituting over 90% of cases among individuals diagnosed with primary liver cancer [3, 4]. Moreover, the occurrence of HCC is linked to numerous etiological risk factors, including but not limited to alcohol abuse, chronic infection with hepatitis, non-alcoholic steatohepatitis (NASH), drug-induced liver injury, autoimmune liver disease, and exposure to aflatoxins [5, 6]. Despite remarkable therapeutic advances, HCC continues to have an unfavorable prognosis, as evidenced by a 5-year survival rate of 15–38% in the USA [7, 8] and in Asia [9]. This unfavorable outcome can be attributed to such factors as chemotherapy resistance, delayed diagnosis, and frequent recurrent and metastatic potential.

Various treatment modalities, including radiofrequency ablation, surgical resection, and transarterial chemoembolization, exhibit potent effectiveness for treating HCC localized in the liver. On the other hand, systemic therapy utilizing diverse drugs that target the tumor microenvironment (TME) proves effective in managing HCC cases that are not amenable to surgical resection. Ever since its initial validation in 2009, this approach has demonstrated promising results in enhancing for improving the survival outcomes of individuals diagnosed with unresectable HCC [10]. The adoption of systemic therapy involving molecularly targeted agents (MTAs) has evolved into a continuously advancing treatment approach for advanced-stage HCC. Furthermore, a range of multi-kinase inhibitors, such as sorafenib, regorafenib, lenvatinib, cabozantinib, and the vascular endothelial growth factor (VEGF) inhibitor ramucirumab, are extensively utilized in the clinical setting [11–14]. Alongside MTAs, immunotherapeutic agents represent the most rapidly expanding drug class and remarkably influence cancer therapy and overall human health [15]. Cancer therapeutics have witnessed significant advancements through the utilization of immunotherapeutics, including immune checkpoint inhibitors (ICIs), chimeric antigen receptor T cells (CAR-T), and bispecific antibodies (BsAb) [16]. Of note, ICIs specifically function by disrupting the interactions between immune checkpoint ligands linked to T-cell exhaustion and their corresponding receptors on tumor cells and antigen-presenting cells (APCs), thereby presenting a new approach for treating individuals diagnosed with HCC [17]. Despite the significant shift in the approach to systemic therapy for HCC, the therapeutic outcomes in cases of advanced HCC are not satisfactory because of the lack of techniques for assessing treatment resistance and predicting treatment response. Given the limited range of available treatment options available for HCC, developing novel prognostic biomarkers and efficient treatment techniques is necessary.

Current research in cancer prevention primarily centers around T lymphocytes and their antigen-directed cytotoxicity [18]. Efforts to enhance clinical responses and identify predictive biomarkers have predominantly centered on T-cell populations [19]. Notably, the utilization of adoptive T-cell (ATC) therapy, which entails the infusion of autologous or allogeneic T cells into diseased individuals, has led to encouraging outcomes in recent times. To foster the growth of engineered lymphocytes, it is vital to undertake in vitro expansion of tumor-specific T cells as it bears substantial implications in this regard [20]. The activity of T-cells is governed by multiple negative regulators, functioning as “checkpoint molecules” [21]. Immunomodulatory antibodies, known as ICIs, are uniquely engineered to selectively interact with molecules involved in immune modulation, like anti-CTLA-4 and anti-PD-1. Multiple official regulatory agencies have granted approval for the utilization of various ICIs in cancer therapy. Additionally, recent investigations found a set of 33 T-cell proliferation-related genes (TRGs) that exhibit the capacity to stimulate the proliferative activity of T cells, enhance the secretion of proinflammatory cytokines, and augment the levels of activation markers [22]. Nonetheless, there remains a marked gap in our comprehension regarding the precise implications of these TRGs on the prognostication and response to medical interventions in the context of HCC.

In this comprehensive investigation, the expression patterns of TRGs were extensively examined to provide a comprehensive understanding of the HCC immunological landscape. Initially, individuals diagnosed with HCC were partitioned into two distinct molecular subtypes by analyzing levels of TRGs. Next, the study participants were further categorized into two clusters as per differentially expressed genes (DEGs). A prognostic and immunotherapy response prediction model was built using TRG scores that incorporated seven predictive genes. Finally, to confirm the predictive capacity of the model, the levels of seven prognostic genes were tested through qRT-PCR in two HCC cell lines alongside one cell line derived from normal liver.

## Material and methods

### Data retrieval and processing

Data pertaining to genetic mutations, gene expression levels measured in fragments per kilobase million (FPKM), and clinical information of individuals diagnosed with HCC were acquired from TCGA. In total, 374 HCC samples and 50 normal samples were included in the exploration. To facilitate data processing, the FPKM values were converted into transcripts per kilobase million (TPM) values by means of Rstudio software (v1.4.1106). In addition to this, a dataset of gene expression (GSE52018) pertaining to liver cancer was retrieved from GEO, available at https://www.ncbi.nlm.nih.gov/geo/. This dataset was then integrated with the TCGA data to enable comprehensive analysis. Clinical information of the individuals, obtained through patient IDs, was cross-referenced with their transcriptomic data. Subsequently, a screening process was conducted as per predefined inclusion criteria, which included the availability of expression profiles and a confirmed histological diagnosis of HCC. For further analysis, data meeting the aforementioned criteria were obtained from both the GSE52018 and TCGA datasets, comprising a cohort of 419 cases. Additionally, the GEO-retrieved GSE10186 dataset was utilized to present external verification.

### Transcriptional and genetic evaluation of TRGs in HCC

The study conducted by Legut et al. [22] provided information regarding the names of genes and their expression patterns for the set of 33 TRGs. To further analyze these TRGs, somatic mutations were depicted on a waterfall plot generated by means of the R package “maftools.” Additionally, data on transcriptional mutations associated with the 33 TRGs were acquired from TCGA (Supplementary Table 1), enabling the assessment of the frequency of copy number variations (CNVs) and the identification of the specific genomic locations. Additionally, the package “limma” was utilized to implement the Wilcoxon signed-rank test, enabling the comparison of TRG levels between tumor and normal tissues. Survival analysis was conducted utilizing the log-rank test to calculate the corresponding *P*-values, while correlation analysis was conducted to illuminate the interplay among the TRGs.

### Cluster analysis for identifying TRG-related molecular subtypes

For the classification of HCC patients based on TRG expression, the R package “ConsensusClusterPlus” was employed, resulting in the formation of two distinct molecular clusters. Moreover, the optimal number of subtypes was determined utilizing the K-means algorithm. Subsequently, the clustering of TRG-related molecular subtypes was achieved through the utilization of the “limma” and “ggplot” R packages by principal component analysis (PCA).

### Evaluation of the biological and clinical characteristics of the two TRG-related molecular clusters

To analyze the prognostic implications of the two TRG-related molecular clusters, a survival analysis was carried out by means of the “survminer” and “survival” packages. Survival time differences between the two clusters were visually represented through the generation of Kaplan–Meier (KM) curves. Additionally, a comprehensive visualization of age, TNM stage, sex, and TRG expression patterns in each cluster was depicted using the "pheatmap" R package through the creation of heatmap. Moreover, the "GSVA" R package was utilized for gene set variation analysis (GSVA), enabling the examination of variations in immune cell infiltration across the clusters. A comprehensive investigation of immune-associated pathways in the two clusters was achieved through the implementation of single-sample gene set enrichment analysis (ssGSEA). The “pheatmap” package was instrumental in visually representing and interpreting these results.

### DEG and enrichment analyses

By utilizing the “limma” R package, DEGs in both TRG-related molecular clusters mentioned earlier were identified. This detection was based on strict criteria, including a threshold of |log FC| values greater than 1 and adjusted *P*-values below 0.05. To explore the potential implication of these DEGs in HCC, Gene Ontology (GO) and Kyoto Encyclopedia of Genes and Genomes (KEGG) analyses were executed. Furthermore, the “ConsensusClusterPlus” R package enabled a more refined understanding of the classification of individuals into two distinct gene clusters on the basis of the expression patterns of DEGs. Lastly, the survival outcomes were compared between the two clusters.

### Development and assessment of the prognostic TRG_score model

A prognostic TRG_score model was created to assess the level of risk in individuals diagnosed with HCC. This model employed various statistical techniques, including Lasso and multivariate Cox regression analyses, as well as cross-validation, using the “glmnet” R package. Using the identified signature genes, a predictive model was developed. This model allowed for the computation of risk scores (RS) (TRG_scores) for each individual. The equation is illustrated below:

$${\text{TRG}}\_{\text{score}} = \Sigma \left( {{\text{Expi}} \times {\text{coefi}}} \right)$$

In the provided equation, the terms Coefi and Expi denote the risk coefficient and expression of each gene, respectively. On the basis of the median RS, individuals were categorized into high-risk group (HRG) and low-risk group (LRG). The interrelationship among such variables as RSs, TRG, and gene clusters was established. Additionally, independent prognostic factors for HCC were identified through the implementation of Cox regression explorations (univariate and multivariate). A characteristic nomogram was developed in accordance with age, tumor stage, sex, and RS for the prediction of survival probabilities at specific time intervals of 1, 3, and 5 years. Moreover, the creation of calibration graphs for the nomogram was facilitated by “survival” and “rms” R packages.

### Study of TME across the HRG and LRG

The relationship between the abundance of immune cells and the expression of the seven prognostic genes was examined. Using the “ESTIMATE” R package, TME scores, including estimate, stromal, and immune scores, were calculated on the basis of gene expression profiles [23]. Afterward, the correlation between cancer stem cell (CSC) index and RSs was examined.

### Exploration of immune checkpoints and immune-related gene predictive index

The “ggpubr” R package was leveraged to comparatively assess the levels of immune checkpoint genes (ICGs) between the HRG and LRG. Furthermore, a multivariate Cox regression analysis was executed to build a T cell proliferation-related model. The objective was to verify the involvement of the prognostic model in determining immunotherapy responsiveness.

### Response to chemotherapeutic drugs

Half-maximal inhibitory concentration (IC_50_) represents the concentration of a drug required to curtail the maligancy of cancer cells by 50%. To ascertain the link between RSs and response to immunotherapeutic and chemotherapeutic agents, the expression of ICGs, immune subtypes, and the IC_50_ values of drugs were examined in the HRG and LRG. This investigation was conducted utilizing the “pRRophetic” R package.

### Expression of the seven prognostic TRGs in normal and HCC cell lines

The measurement of the mRNA levels of the seven prognostic TRGs was completed by qRT-PCR. This analysis involved one normal liver cell line (referred to as the “normal group”) and two HCC cell lines (referred to as the “HCC group”). Besides, a comparison was made for the levels of key TRGs between HCC and normal groups utilizing the Human Protein Atlas (HPA) database.

## Results

### Transcriptional and genetic alterations in TRGs in HCC

**Fig. 1:**
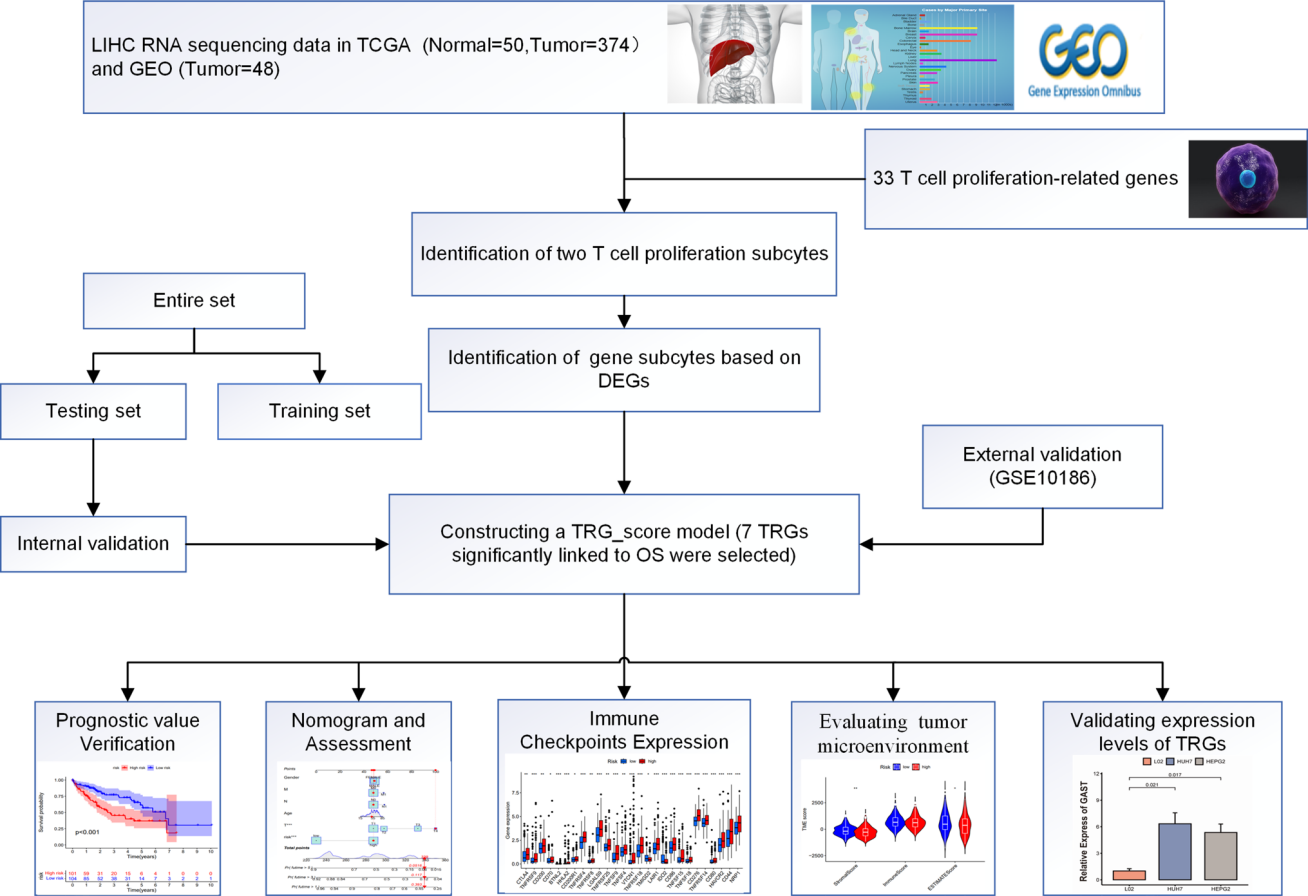
Flow diagram illustrating the research design

**Fig. 2:**
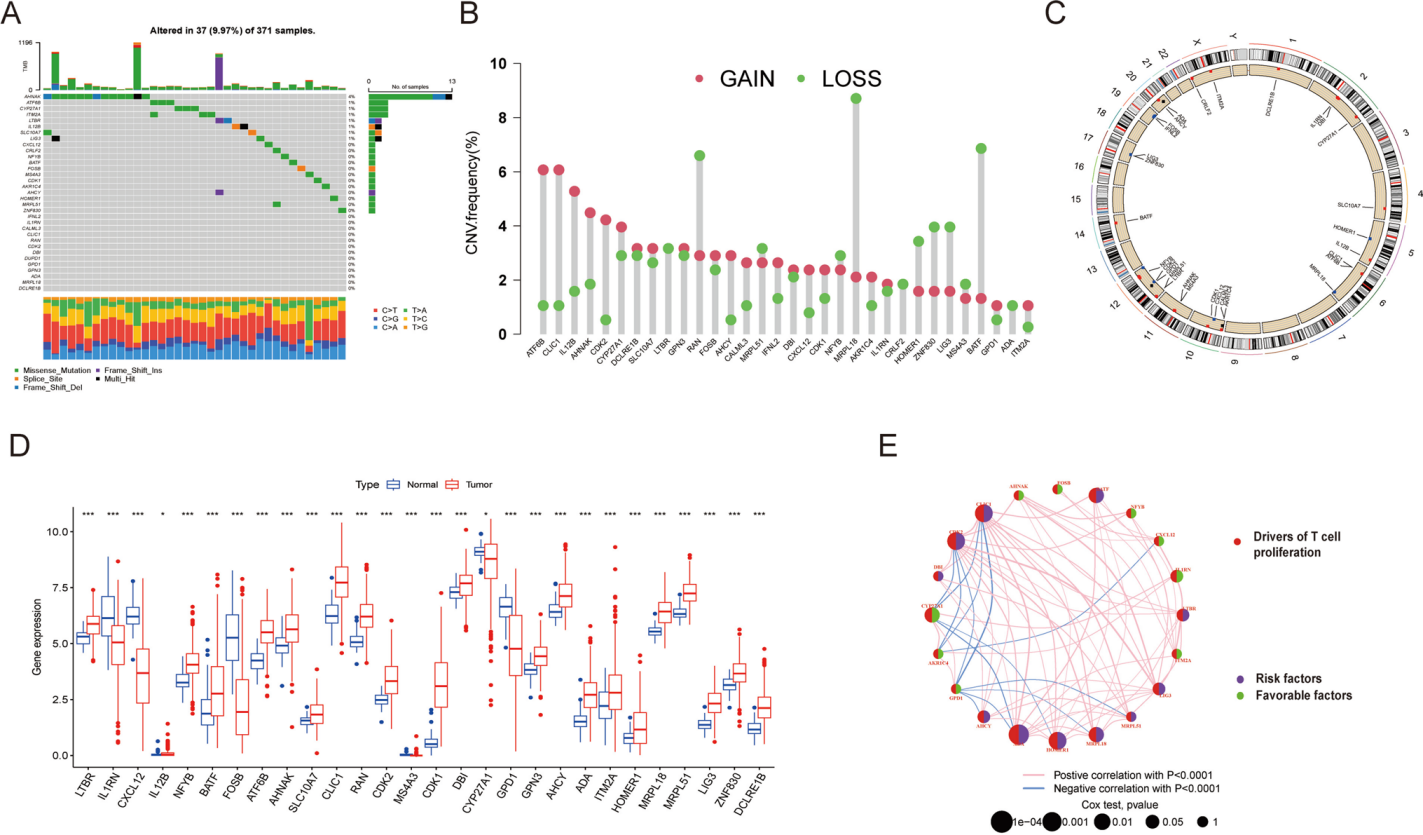
Genetic and transcriptional variations in 33 TRGs in HCC. **A** Mutation frequencies of 33 TRGs in individuals diagnosed with HCC in the TCGA cohort. **B** Frequencies of copy number amplification, copy number deletion, and non-CNV in TRGs. **C** Chromosomal location and alterations in T cell proliferation regulators. **D** Relative expression levels of TRGs that exhibited differential expression between tumor and healthy samples. **E** A comprehensive network is constructed to visualize and analyze the intricate landscape of interactions among TRGs, with the connecting lines representing their interplay. Associations are denoted by red and blue colors, signifying negative and positive associations. The significance levels of the *P*-values are denoted by the symbols “ns,” “*,” “**,” and “***,” which signify “not significant,” “*P* < 0.05,” “*P* < 0.01,” and “*P* < 0.001,” respectively

The study protocol is illustrated in Fig. 1. A comprehensive analysis was conducted on a set of 33 TRGs, wherein somatic mutations were analyzed to screen for common variants of TRGs in the context of HCC. As depicted in Fig. 2A, 37 (9.97%) somatic mutations were detected among 371 individuals diagnosed with HCC. Among these mutations, *AHNAK* manifested the highest mutation rate (4%), whereas the remaining 7 TRGs, namely *ATF6B, CYP27A, ITM2A, LTBR, IL12B, SLC10A7,* and *LIG3*, shared a mutation rate of 1%. Subsequently, the presence of CNVs was assessed. The analysis revealed high occurrence of CNVs was found to be high in *ATF6B, CDK2, CLIC1, AHNAK*, and *IL12B*, while *BATF, RAN, ZNF830, MRPL18, LIG3*, and *HOMER1* exhibited low frequency of CNVs (Fig. 2B). Additionally, Fig. 2C displays the chromosomal location of CNVs affecting TRGs on corresponding chromosomes. Overall, these findings imply that multiple TRGs undergo mutations in individuals diagnosed with HCC.

In the HCC and normal tissues, a total of 28 notably differentially expressed TRGs were singled out, as shown in Fig. 2D. This investigation indicates that the levels of TRG differ between patients and healthy subjects. Simultaneously, the T cell proliferation network illustration (Fig. 2E) presents a comprehensive depiction of the interplay among TRGs, the relationships among influential factors, and their substantial prognostic value in individuals diagnosed with HCC.

### Exploration of two TRG-related molecular clusters

**Fig. 3:**
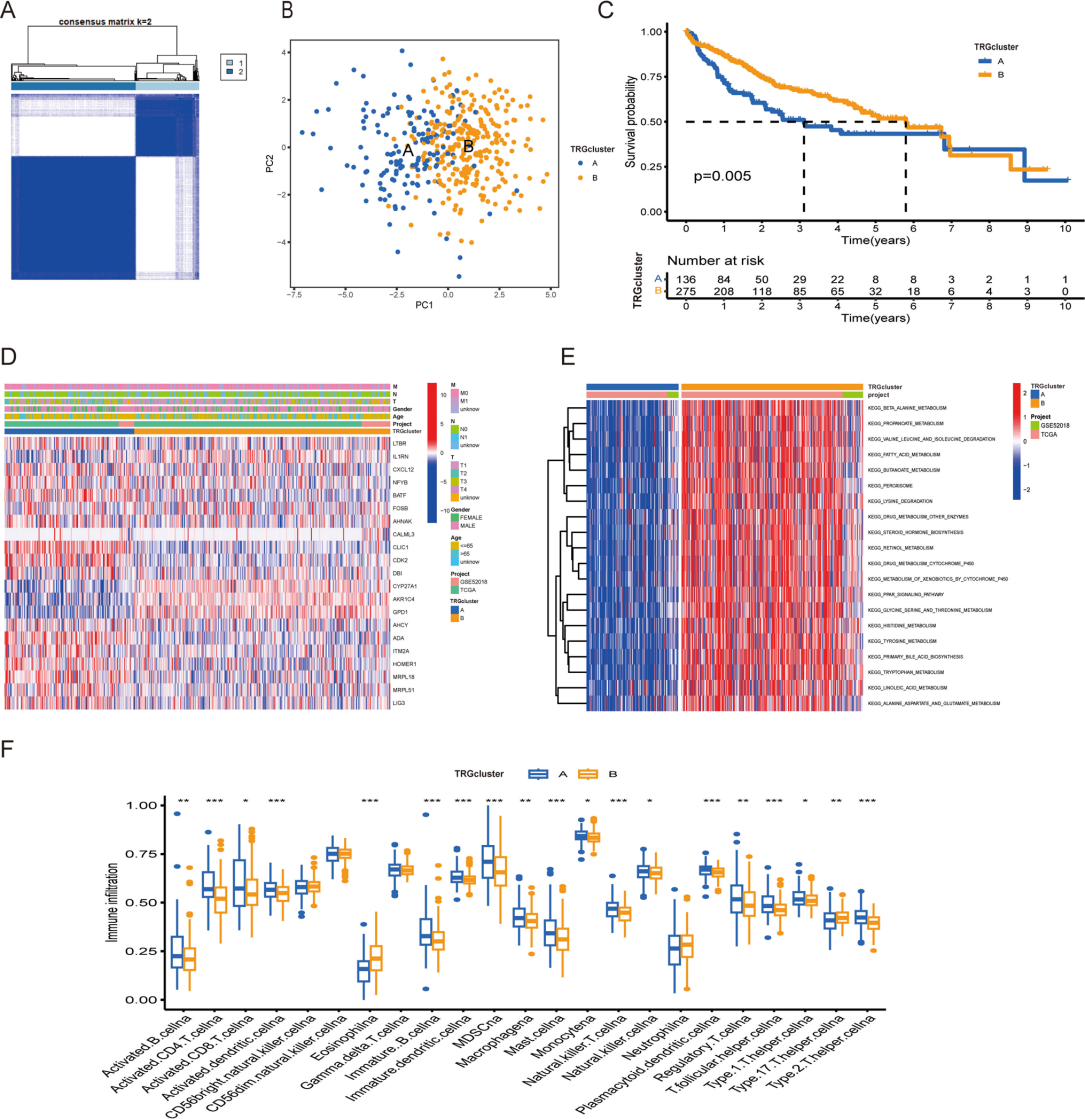
TRG clusters in HCC samples along with the clinical characteristics, TME across these clusters. **A** Two molecular clusters associated with TRGs were characterized via consensus clustering exploration. **B** KM curve visually presenting significant variations (*P* = 0.005) in survival time across the two identified clusters. **C** PCA plot visually presenting variations across the two identified clusters. **D** Heatmap illustrating variations in clinical characteristics and TRG expression across the two clusters. **E** GSVA showing the enriched cascades in the molecular clusters, where blue and red depict inhibited and activated cascades, respectively. **F** ssGSEA reported variations in the infiltration of immune cells across the two identified clusters

To comprehend the influence of TRGs on the survival outcomes of individuals diagnosed with HCC, a consensus clustering algorithm was leveraged for the classification of individuals into two molecular clusters according to the expression of the 33 TRGs (Fig. 3A). The analysis revealed that a k-value of 2 was identified as optimal, resulting in the division of clusters A (n = 141) and B (n = 278). PCA suggested noteworthy differences in the levels of TRGs between the two identified clusters (Fig. 3B). KM survival analysis (Fig. 3C) established that individuals belonging to cluster B exhibited better survival outcomes relative to those of cluster A (*P* = 0.005). Moreover, the interplay among clinical characteristics, molecular clusters associated with TRGs, and TRG expression was visually presented in a heatmap, as depicted in Fig. 3D. GSVA reported considerable enrichment of such critical biological pathways as metabolism, cytochrome P450, and hormone biosynthesis in cluster B (Fig. 3E). The outcomes of ssGSEA established that infiltration of immune cells was enhanced in the cluster A of molecular subtype relative to cluster B (Fig. 3F). These outcomes depict that the prognosis and certain clinical characteristics differ across the two molecular clusters. Consequently, DEGs across the two clusters were detected to gain deeper insights into the distinctive molecular features of the two clusters.

### Identification of two distinct gene clusters as per the DEGs

**Fig. 4:**
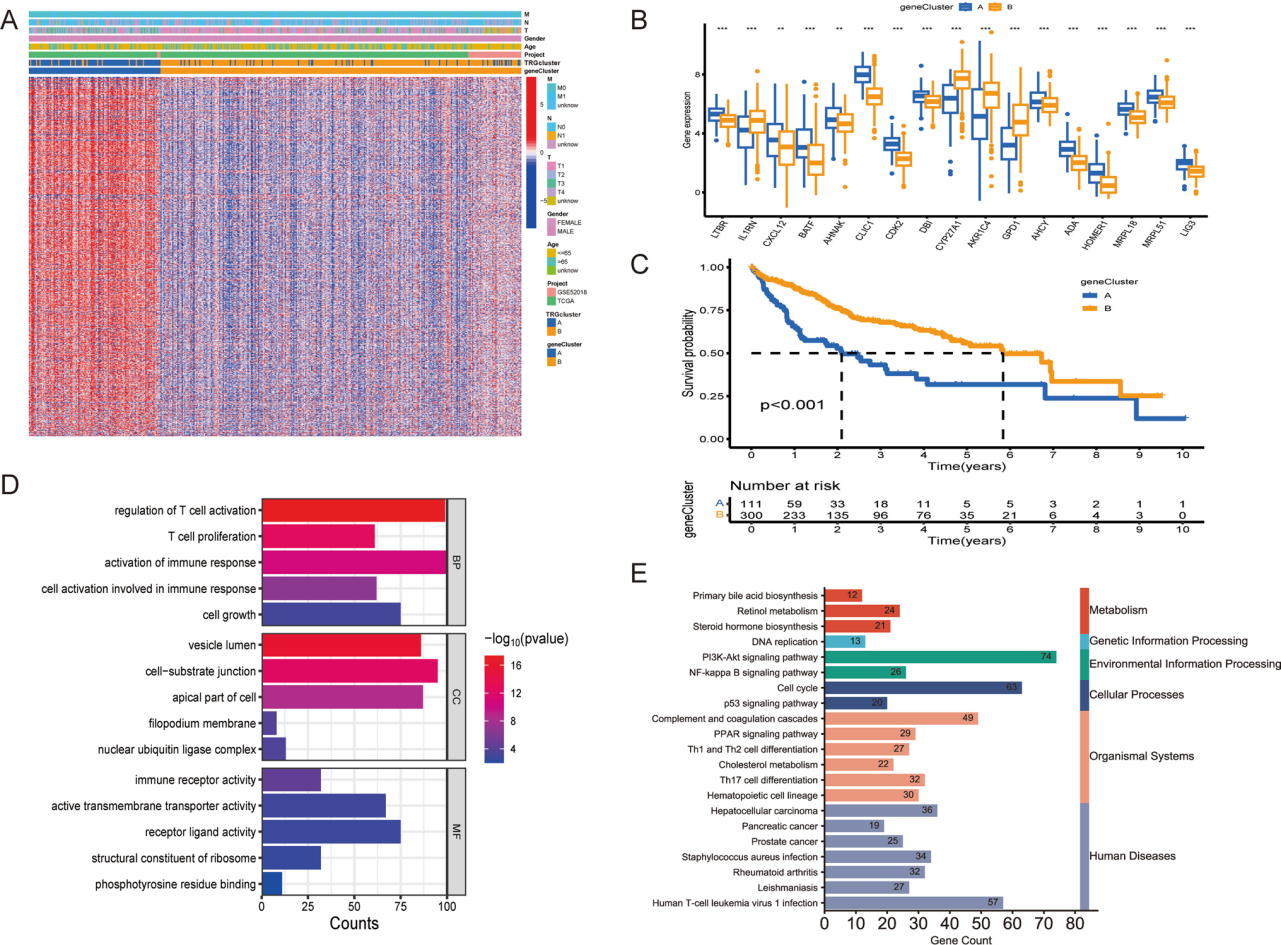
Establishment of gene clusters on the basis of DEGs identified. **A** A visual representation in the form of a heatmap illustrating the relationship between clusters and various clinical characteristics. **B** Expression patterns of DEGs across the identified gene clusters. **C** KM curve depicting prolonged survival time in individuals classified under cluster B (*P* < 0.001). **D**, **E** Most significant or shared GO terms and KEGG pathways. The significance levels of the *P*-values are denoted by the symbols “ns,” “*,” “**,” and “***,” which signify “not significant,” “*P* < 0.05,” “*P* < 0.01,” and “*P* < 0.001,” respectively

In total, 2049 DEGs were characterized within the two molecular clusters associated with TRGs. Subsequent univariate Cox regression investigation revealed that 1009 out of these DEGs (*P* < 0.05) were linked to the prognostic implication, specifically the overall survival (OS), of individuals diagnosed with HCC. Using the expression patterns of prognostic DEGs, a consensus clustering algorithm enabled the classification of individuals diagnosed with HCC into two distinct gene clusters (clusters A and B). Figure 4A illustrates the association among clinical characteristics, molecular clusters associated with TRGs, gene clusters, and DEGs. Variations in the expression of prognostic TRGs across the gene clusters are visually presented as a boxplot in Fig. 4B. KM curves (Fig. 4C) established a significantly enhanced OS (*P* < 0.001) in gene cluster B relative to gene cluster A, as evidenced by log-rank test.

GO analysis established that the prognostic DEGs were primarily enriched in biological processes (BPs) such as regulation of T-cell activation, cell growth, immune response activation, and T-cell proliferation. In terms of cellular components (CCs), the enrichment was observed in locations such as the vesicle lumen, cell-substrate junction, and the apical part of the cell. Moreover, molecular functions (MFs) of these DEGs encompassed activities such as receptor-ligand activity, active transmembrane transporter activity, and immune receptor activity. KEGG analysis demonstrated that the prognostic DEGs were implicated in various pathways. These pathways encompassed primary bile acid biosynthesis, complement and coagulation cascades, and HCC-related pathways (Fig. 4D, E).

### Development and validation of a TRG_score model

**Fig. 5:**
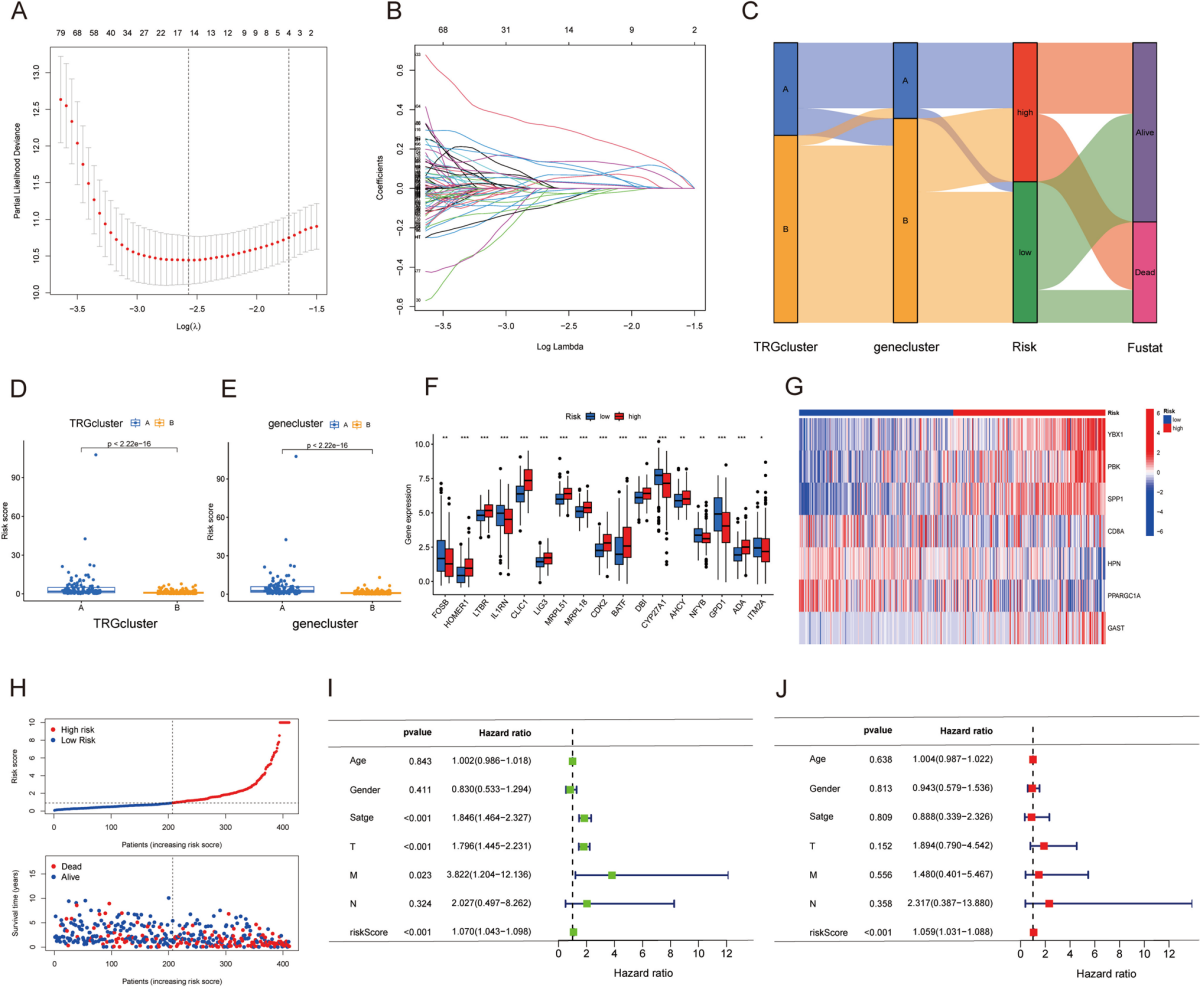
Development of a prognostic model for HCC. **A**, **B** Key TRGs were subjected to analyses using LASSO regression and partial likelihood deviance. **C** A comprehensive Sankey diagram depicting an interconnected representation of distributions of TRG subtypes, risk groups, gene subtypes, and survival status. **D**, **E** Variations in RSs across the two molecular clusters and the two gene clusters. **F** Variations in the patterns of TRG expression across the HRG and LRG. **G** A visual representation in the form of a heatmap depicting the levels of seven prognosis-predictive genes in the HRG and LRG. **H** RSs and survival outcomes of individual samples. **I**, **J** Forest plots presenting analyses of univariate and multivariate Cox regression in individuals diagnosed with HCC

Considering the connection between the DEGs characterized in the two molecular clusters associated with TRGs and patient survival, a model was developed to predict prognostic implications utilizing these genes. Through the implementation of LASSO and multivariate Cox regression analyses, seven genes were identified as predictive factors in this model. These genes include *YBX1, PBK, SPP1, CD8A, HPN, PPARGC1A,* and *GAST*. Following this, the identified genes were leveraged to generate the TRG_score model. The methodology employed for LASSO regression analysis is visually presented in Fig. 5A, B. The RS was computed per the TRG_score model utilizing the following mathematical formula:

$$\begin{gathered} {\text{RS}} = \left( {0.{5478} \times {\text{expression}}\;{\text{of}}\;{\text{YBX1}}} \right) + \left( {0.{2372} \times {\text{expression}}\;{\text{of}}\;{\text{PBK}}} \right) \hfill \\ \quad \quad + \left( {0.{1175} \times {\text{expression}}\;{\text{of}}\;{\text{SPP1}}} \right) + \left( {0.{2879} \times {\text{expression}}\;{\text{of}}\;{\text{GAST}}} \right) \hfill \\ \quad \quad + \left( { - 0.{2775} \times {\text{expression}}\;{\text{of}}\;{\text{CD8A}}} \right) + \left( { - 0.{1839} \times {\text{expression}}\;{\text{of}}\;{\text{HPN}}} \right) \hfill \\ \quad \quad + \left( { - 0.{2453} \times {\text{expression}}\;{\text{of}}\;{\text{PPARGC1A}}} \right) \hfill \\ \end{gathered}$$

A Sankey diagram was generated to visually present the categorization of individuals diagnosed with HCC into the two TRG-associated molecular clusters, two gene clusters, and two RS groups (based on the median RS) (Fig. 5C). The distribution of RSs in the two molecular (Fig. 5D) and gene (Fig. 5E) clusters were visualized on boxplots. Figure 5F depicts the differential expression of TRGs across the HRG and LRG, while Fig. 5G depicts a heatmap displaying the expression patterns of seven specific DEGs across the HRG and LRG (Fig. 5G). It was observed that individuals diagnosed with HCC and having low RSs experienced improved OS, as displayed in Fig. 5H. Moreover, Cox regression analyses, including univariate (Fig. 5I; *P* < 0.001) and multivariate (Fig. 5J; *P* < 0.001), emphasized that the tumor stage and RS served as independent prognostic factors for HCC.

**Fig. 6:**
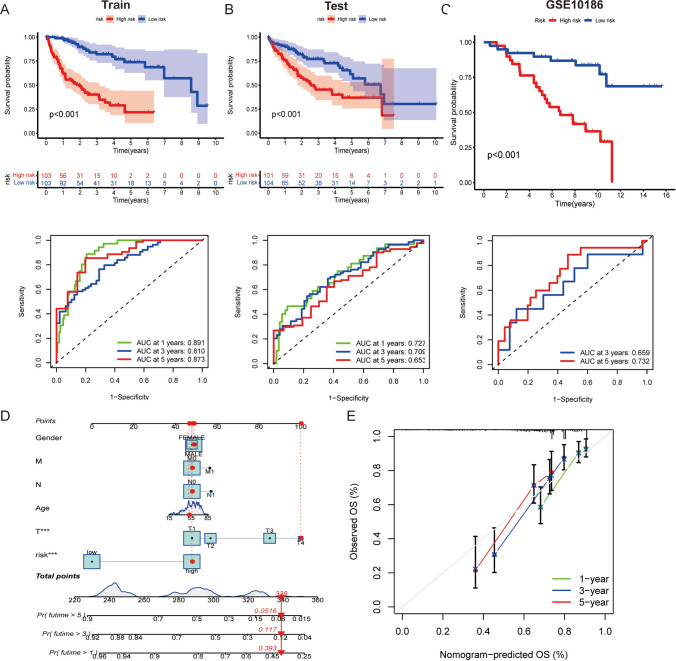
Reliability and effectiveness of the RSs in prognosticating diverse survival results experienced by patients. **A**–**C** Graphical representations of training, internal validation, and external validation sets analyses using KM and ROC curves for prognostic significance of the RSs. **D** Nomogram depicting the prognosis-predictive value of clinicopathological parameters and RSs. **E** Calibration curves depicting the specificity and accuracy of the nomogram

The predictive accuracy of RSs in survival outcomes for HCC cases was ascertained utilizing KM curves and area under receiver operating characteristic (ROC) curves (AUC). The findings highlighted a notable link between low RSs and prolonged survival in both training sets (*P* < 0.001, with AUC values of 0.891, 0.810, and 0.873 for 1-, 3-, and 5-year periods, respectively) (Fig. 6A) and internal validation set (*P* < 0.001, with AUC values of 0.727, 0.709, and 0.653 for 1-, 3-, and 5-year periods, respectively) (Fig. 6B). Due to the limited number of samples (only two) with a survival time of ≤ 1 year in the external validation set in GSE10186, survival probabilities were estimated the 3- and 5-year periods. Moreover, the data underscored a notable link between low RSs and extended survival in the external validation set (*P* < 0.001, with AUC values of 0.659 and 0.732 for 3- and 5-year periods) (Fig. 6C). Overall, these results indicate that RS can act as a reliable predictor of survival in individuals diagnosed with HCC. Furthermore, a nomogram was constructed by integrating RSs with such clinical characteristics as age, sex, and tumor stage, which enabled the prediction of survival probabilities at 1-, 3-, and 5-year time points (Fig. 6D). Remarkably, the calibration curve demonstrated a strong concordance between the predicted and observed OS rates, indicating that the nomogram effectively predicted survival (Fig. 6E).

### Assessment of TME and CSC index across HRG and LRG

**Fig. 7:**
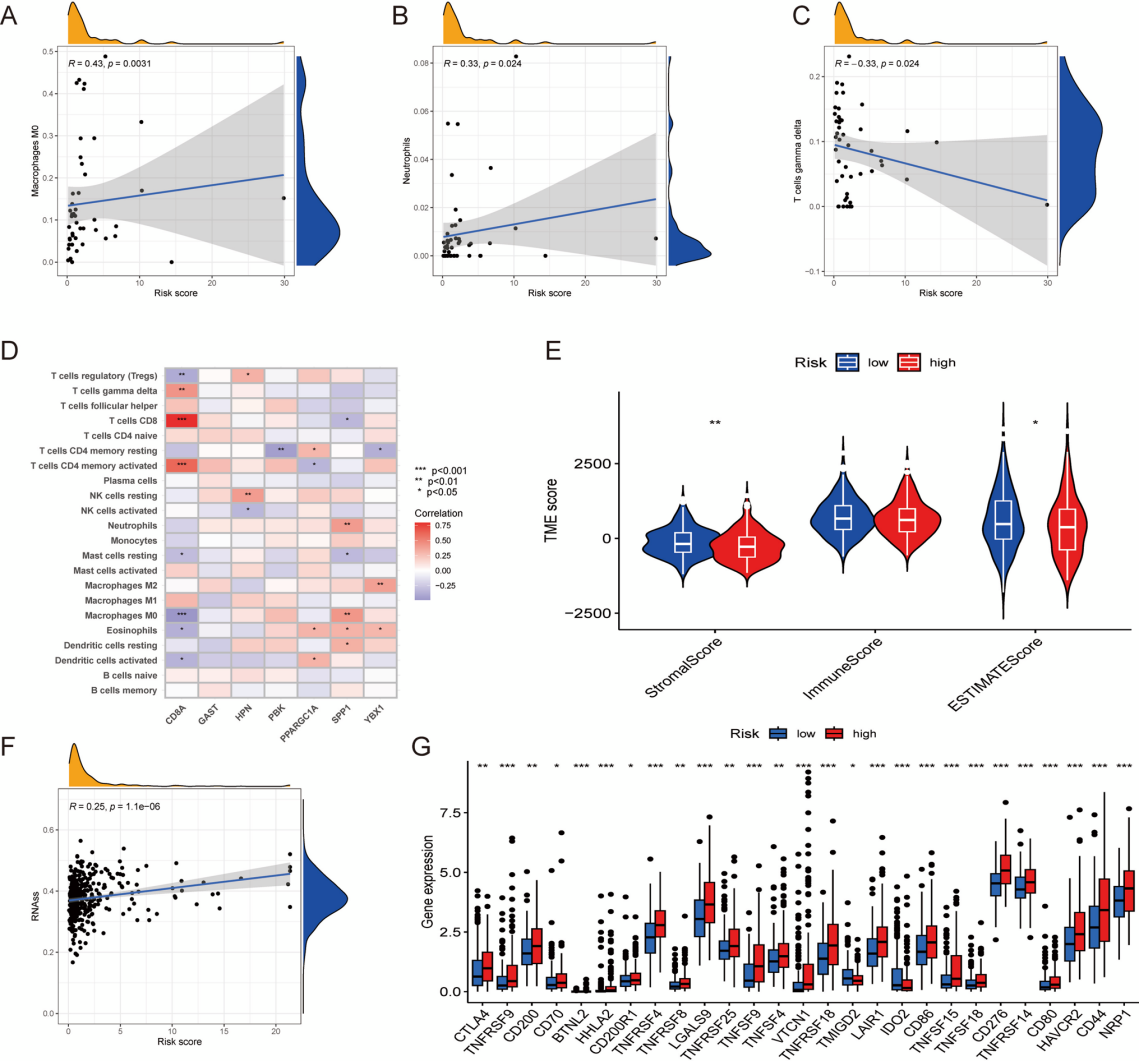
Assessment of TME in the HRG and LRG. **A**–**C** Link between RSs and the abundance of various immune cell populations. **D** Link between the abundance of immune cells and levels of seven prognostic TRGs. **E** Link between risk and TME scores. CSC index **F** in the HRG and LRG is illustrated. **G** Differences in expression of ICGs across the HRG and LRG. The significance levels of the *P*-values are denoted by the symbols “ns,” “*,” “**,” and “***,” which signify “not significant,” “*P* < 0.05,” “*P* < 0.01,” and “*P* < 0.001,” respectively

Following the stratification of individuals into two distinct risk groups (HRG and LRG), the predictive accuracy of the risk model was ascertained through bioinformatic tools. The association between RSs and the abundance of tumor-infiltrating immune cells (TIICs) was visually presented on scatter plots (Fig. 7A–C). An inverse correlation was observed between RS and the abundance of gamma delta T cells, activated M0 macrophages, and neutrophils. Furthermore, the relationship between the levels of seven prognostic genes and the abundance of TIICs is illustrated in Fig. 7D. A remarkable link was witnessed between the abundance of most immune cells and levels of all prognosis-predictive genes except for *GAST*. Additionally, low-RSs were linked to high stromal scores(Fig. 7E). Figure 7F presents a graphical representation of a significant correlation between the CSC index and the RS (R = 0.25, *P* < 0.001).

### Expression of ICGs in the HRG and LRG

Figure 7G illustrates that the HRG manifested a significant upregulation of the expression of the majority of ICGs (*P* < 0.05). This finding underscores the potential benefits of ICI therapy for individuals who have been diagnosed with HCC and exhibit high RSs.

### Drug sensitivity analysis

**Fig. 8:**
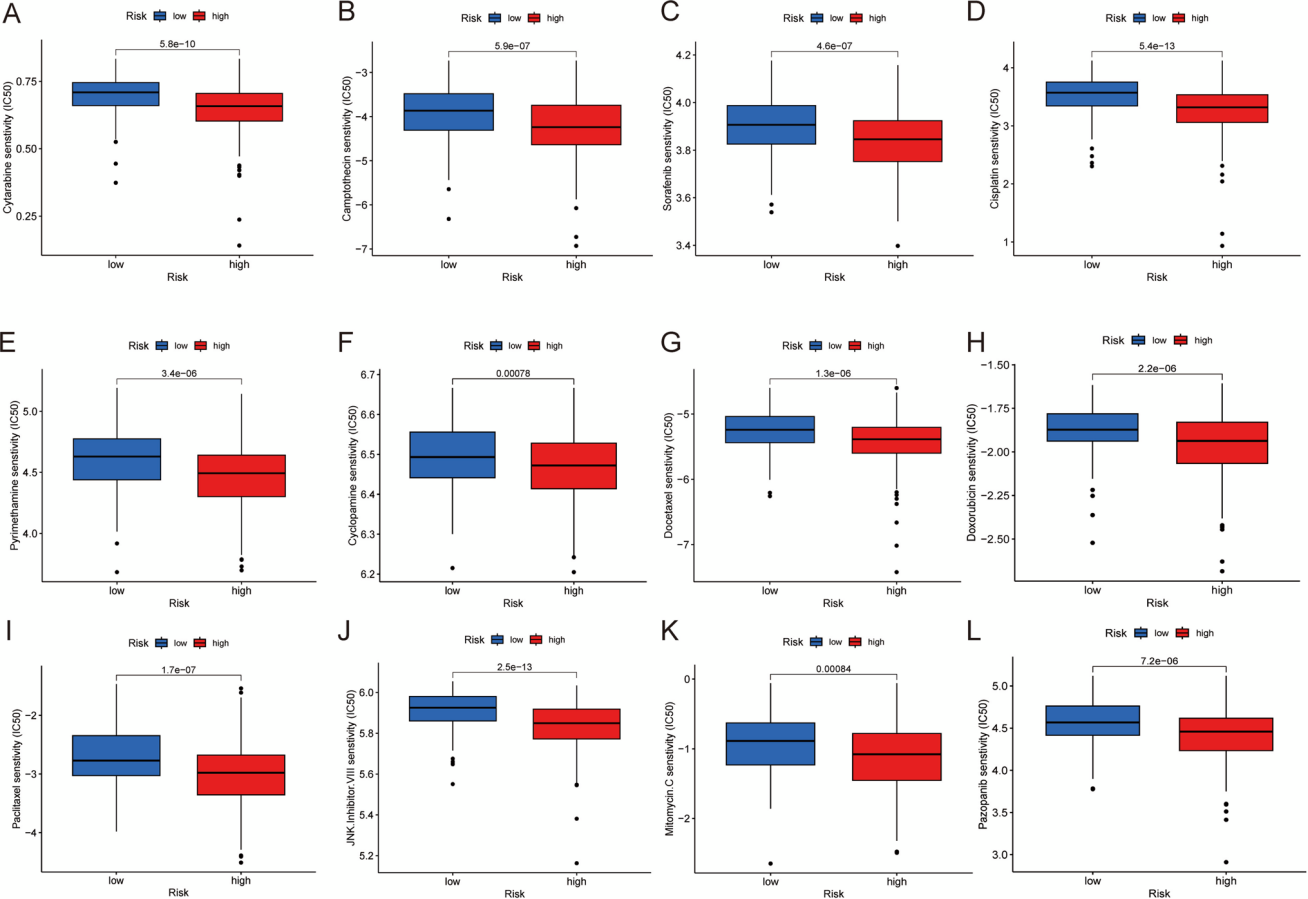
Analysis of drug sensitivity. **A**–**L** Drugs with significantly varying IC_50_ values across the HRG and LRG

A comparison was made to evaluate the sensitivity of individuals to specific chemotherapeutic agents across HRG and LRG. The findings depicted that individuals classified in LRG had higher IC_50_ values for sorafenib, cytarabine, and camptothecin. This observation implies a potential link between the seven TRGs and drug susceptibility (Fig. 8A–L).

### Comparison of the levels of the seven prognostic genes in HCC and normal liver cells

**Fig. 9:**
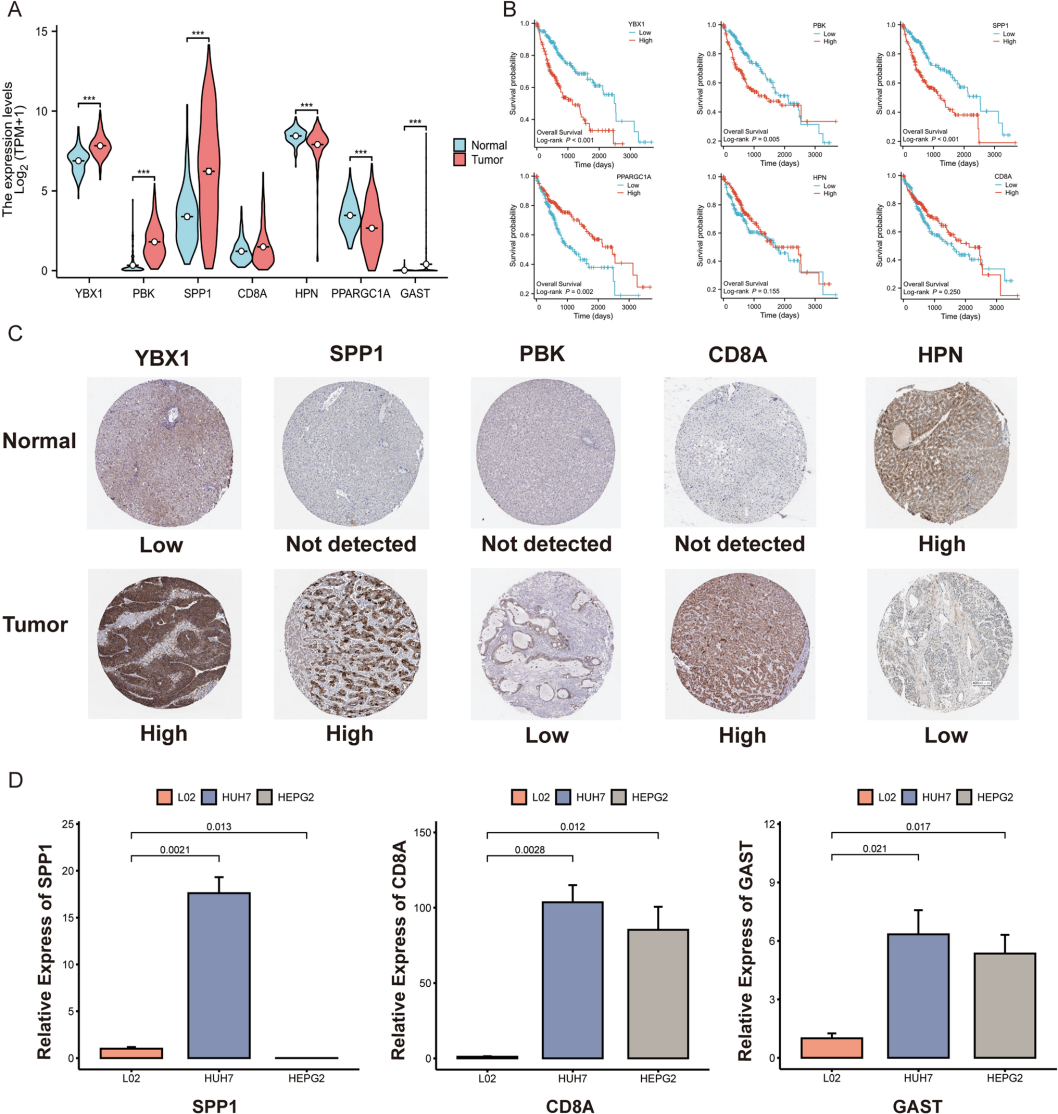
Levels of prognostic TRGs in HCC and normal cells and in vitro analysis. **A** Differential expression of the seven prognostic TRGs in normal and tumor tissues in TCGA and GEO datasets. **B** KM analysis showing the prognostic value of the seven TRGs in the TCGA dataset. **C** IHC data from the HPA database demonstrating variations in the protein levels of five prognostic TRGs between tumor and normal tissues. **D** qRT-PCR detection of the mRNA levels of *SPP1, CD8A*, and *GAST* in normal liver cells and HCC cell lines

Figure 9A presents a violin plot depicting the contrasting expression patterns of the seven prognostic genes that predict prognosis in individuals with HCC compared to healthy subjects. Through KM analysis, it was observed in Fig. 9B that individuals who exhibited low expressions of *YBX1, PBK*, and *SPP1* showed a significant extension in OS time. Furthermore, the protein levels of five genes that predict prognosis were examined in HCC tissues in comparison to non-tumor tissues by means of immunohistochemical (IHC) data from the HPA database (Fig. 9C). To ascertain the reliability of the 7-gene prognostic model, qRT-PCR was executed to assess the levels of the seven prognostic genes in HCC and normal liver cells. The findings reported that the expression of three signature genes (*SPP1, CD8A*, and *GAST)* varied significantly between HCC and normal liver cells (Fig. 9D).

## Discussion

HCC is considered one of the most perilous malignancies across the globe [24, 25]. The highly heterogeneous nature of HCC, combined with its atypical early symptom presentation, leads to a significant number of individuals receiving a diagnosis at an advanced stage. Consequently, many of these individuals are unable to undergo radical procedures as a viable treatment option [26]. In recent years, the treatment landscape for HCC has undergone diversification due to the establishment of ICI-based therapies as a standard of care and the emergence of various systemic therapies [27, 28]. Nevertheless, the optimal sequencing of drug administration remains undetermined, and the identification of biomarkers can effectively predict treatment response and efficacy [28]. In this study, a TRG-based prognostic model was developed for predicting HCC prognosis, followed by screening for potential therapeutic targets to enhance patient outcomes.

The prominent significance of T cells in the field of tumor immunotherapy cannot be underestimated. Nevertheless, the current body of research has predominantly centered on unraveling the mechanistic actions deployed by T cells to effectively eliminate tumors or has been limited to studying a specific subset of immune-related cells [29, 30]. As a consequence, the outcomes of these investigations fail to capture the collective significance of TRGs. In contrast, this research addressed the genetic and transcriptional alterations in TRGs in individuals diagnosed with HCC.

This study involved the categorization of individuals diagnosed with HCC from the TCGA and GSE52018 datasets into molecular clusters as per the expression of the 33 TRGs. Furthermore, it was uncovered that these TRGs had significant implications for prognostic assessment for patients. TME characteristics, represented by stromal and estimate scores, exhibited notable differences among the molecular clusters associated with specific TRGs, thereby establishing a link between TME and TRGs. The TME is comprised of various components, including immune and inflammatory cells, the extracellular matrix (ECM), and secreted cytokines. These components collectively influence and facilitate the advancement of cancer growth [31–33]. Moreover, there exists a correlation between immune cells in TME and carcinogenesis [34]. In the context of HCC, immune cell populations, such as Treg cells, granulocyte monocyte progenitors, hematopoietic stem cells, and multipotent progenitor cells, exhibit abnormalities [35], suggesting the potential suitability of immunotherapy interventions. Moreover, through the implementation of functional enrichment analyses in the two molecular clusters, it was revealed that the transcriptomic alterations in TRGs were remarkably linked to immune-associated biological cascades.

In addition, individuals diagnosed with HCC were stratified into two gene clusters on the basis of the DEGs identified within the molecular clusters. Notably, a substantial disparity in survival rates was observed between the two gene clusters. In light of these findings, the DEGs identified across the two clusters hold the potential to serve as prognostic indicators for predicting the prognosis of individuals diagnosed with HCC in clinical settings. Furthermore, a prognostic TRG_score model was built in accordance with the seven TRGs, and its accuracy in predicting outcomes was validated using both internal and external datasets. To evaluate the significance of the model, qRT-PCR was conducted to examine the levels of the seven prognosis-related TRGs in HCC as compared to normal liver cells. The findings depicted notable variations in the expression of the three genes between HCC and normal liver cells, thereby confirming the reliability of the predictive model. Additionally, a nomogram was built on the basis of the RS and clinical variables. Furthermore, to confirm the robustness of the RS, HCC cases were stratified into HRG and LRG according to the median RS. Of interest, individuals with distinct RSs exhibited notable differences in clinical characteristics, mutations, prognoses, TME attributes, expression of ICGs, RNA levels, and drug sensitivity. Additionally, IHC data acquired from the HPA database established distinct expression patterns of five prognostic genes between HCC and normal tissues.

The challenging task of developing personalized therapies for individuals with HCC is attributed to the multitude and complexity of diverse pathological mechanistic actions [25]. Nonetheless, the field of HCC treatment has experienced significant advancements primarily due to an enhanced comprehension of biological procedures and unprecedented innovations, particularly in the realm of systemic therapy. In the current landscape of HCC treatment, there are several new agents accessible for first- and second-line therapies, which encompass combinations of ICIs [36]. The application of ICIs has remarkably revolutionized HCC therapy, leading to prolonged survival outcomes in individuals who exhibit a positive response [37]. Mechanistically, ICIs function by attenuating the inhibitory signals on T cells, thereby eliciting immune system activation and fostering potent antitumor immune responses [38]. Despite leaps in systemic therapy, HCC prognosis continues to be unfavorable owing to challenges such as drug resistance and frequent recurrence and metastasis [39, 40]. Moreover, the findings of this research may contribute novel perspectives and understanding to the field of HCC treatment.

In this study, the levels of various major ICGs were comparatively evaluated across the risk groups. The outcomes established that the HRG exhibited upregulated expression of ICGs and improved survival, suggesting the potential benefits of ICI therapy for individuals with high RSs. Hence, it can be inferred that the expression patterns of ICGs may serve as a useful marker for assessing the effectiveness of immunotherapy in individuals affected by HCC. The TME encompasses a diverse array of components, including lymphatics, stromal fibroblasts, blood vessels, infiltrating immune cells, and noncellular components like ECM [41]. The components of TME may represent reliable biomarkers that can indicate sensitivity to ICIs. Immune-modulating cells, such as Treg and tumor-associated macrophages, create an immunosuppressive environment that exhibits limited responsiveness to ICIs [42, 43]. In this research, individuals classified in the HRG were found to exhibit low TME scores. This finding highlights the reliability of the prognostic model in evaluating risk from the perspective of TME characteristics in clinical settings.

In light of the unsatisfactory drug response rates and heterogeneity among HCC cases [44], a comprehensive analysis was made to examine drug sensitivity. The findings highlighted that individuals classified in HRG showcased heightened sensitivity towards several different chemotherapeutic drugs, such as cytarabine, sorafenib, and camptothecin. This revelation may offer additional therapeutic drug options available for the treatment of HCC. Additionally, qRT-PCR was carried out to ascertain the expression of the seven genes associated with prognosis prediction in two HCC cell lines and one normal liver cell line. The findings highlighted notable variations in the levels of *SPP1*, *CD8A*, and *GAST* between HCC and normal cells, which is concordant with the outcomes of differential expression analysis in publicly available databases, specifically TCGA and GEO. Furthermore, previous studies have reported a certain relationship between these genes and the occurrence and development of HCC [45–50]. Simultaneously, KM analysis depicted that OS differed for SPP1 between tumor and normal samples, thereby suggesting that SPP1 can be used as a new therapeutic target against HCC.

Several limitations of this study need to be acknowledged. It is characterized by a retrospective design, and all bioinformatic investigations rely on data from publicly accessible databases, which may introduce inherent biases. Therefore, it is imperative to verify the findings of this study through further in vitro or in vivo research. Such efforts can contribute to an enhanced comprehension of the mechanistic basis driving HCC progression and the involvement of TRG in this context. Additionally, the function of SPP1 in HCC was only preliminarily validated through a limited number of in vitro experiments and warrants further investigation. Moreover, the absence of critical clinical data, for example, surgical outcomes and responses to chemotherapy, could not be accessed, potentially impacting the precision and reliability of the obtained results.

To summarize, a reliable prognosis-predictive model was developed utilizing TRGs, enabling accurate prediction of the prognosis of individuals with HCC. Furthermore, the identification of seven signature genes further adds to our understanding of their significant role in determining the HCC prognosis.

### Supplementary Information

Below is the link to the electronic supplementary material.

Supplementary file1 (XLS 20 KB)

## Data Availability

All available data are presented within the article.

## Abbreviations

APCs: Antigen-presenting cells
ATC: Adoptive T-cell
CAR-T: Chimeric antigen receptor T cells
CNV: Copy number variation
DEGs: Differentially expressed genes
ECM: Extracellular matrix
GEO: Gene Expression Omnibus
GO: Gene Ontology
GSVA: Gene set variation analysis
HCC: Hepatocellular carcinoma
ICIs: Immune checkpoint inhibitors
KEGG: Kyoto Encyclopedia of Genes and Genomes
LASSO: Least absolute shrinkage and selection operator
MTAs: Molecularly targeted agents
NASH: Non-alcoholic steatohepatitis
OS: Overall survival
PCA: Principal component analysis
RS: Risk score
ssGSEA: Single-sample gene set enrichment analysis
TCGA: The Cancer Genome Atlas
TME: Tumor microenvironment
TRGs: T-cell proliferation-related genes
VEGF: Vascular endothelial growth factor
